# Face Validity of Observed Meal Patterns Reported with 7-Day Diet Diaries in a Large Population-Based Cohort Using Diurnal Variation in Concentration Biomarkers of Dietary Intake

**DOI:** 10.3390/nu14020238

**Published:** 2022-01-06

**Authors:** Marleen A. H. Lentjes, Linda M. Oude Griep, Angela A. Mulligan, Scott Montgomery, Nick J. Wareham, Kay-Tee Khaw

**Affiliations:** 1 Department of Public Health and Primary Care, University of Cambridge, Cambridge CB1 8RN, UK; angela.mulligan@mrc-epid.cam.ac.uk (A.A.M.); kk101@medschl.cam.ac.uk (K.-T.K.); 2 Clinical Epidemiology and Biostatistics/Nutrition Gut Brain Interactions Research Centre, School of Medical Sciences, Örebro University, 70182 Örebro, Sweden; scott.montgomery@oru.se; 3 MRC Epidemiology Unit, Institute of Metabolic Sciences, University of Cambridge, Cambridge CB2 0QQ, UK; Linda.OudeGriep@mrc-epid.cam.ac.uk (L.M.O.G.); nick.wareham@mrc-epid.cam.ac.uk (N.J.W.); 4 NIHR Biomedical Research Centre, Diet, Anthropometry and Physical Activity (DAPA) Group, MRC Epidemiology Unit, University of Cambridge, Cambridge CB2 0QQ, UK; 5 Clinical Epidemiology Division, Department of Medicine, Solna, Karolinska Institute, 17177 Stockholm, Sweden; 6 Department of Epidemiology and Public Health, University College London, London WC1E 6BT, UK; 7 School of Clinical Medicine/Clinical Gerontology, University of Cambridge, Cambridge CB1 8RN, UK

**Keywords:** meal pattern, eating pattern, diurnal variation, biomarkers, reporting bias, chrono-nutrition, cohort study

## Abstract

In a cross-sectional analysis of a population-based cohort (United Kingdom, *N* = 21,318, 1993–1998), we studied how associations between meal patterns and non-fasting triglyceride and glucose concentrations were influenced by the hour of day at which the blood sample was collected to ascertain face validity of reported meal patterns, as well as the influence of reporting bias (assessed using formula of energy expenditure) on this association. Meal size (i.e., reported energy content), mealtime and meal frequency were reported using pre-structured 7-day diet diaries. In ANCOVA, sex-specific means of biomarker concentrations were calculated by hour of blood sample collection for quartiles of reported energy intake at breakfast, lunch and dinner (meal size). Significant interactions were observed between breakfast size, sampling time and triglyceride concentrations and between lunch size, sampling time and triglyceride, as well as glucose concentrations. Those skipping breakfast had the lowest triglyceride concentrations in the morning and those skipping lunch had the lowest triglyceride and glucose concentrations in the afternoon, especially among acceptable energy reporters. Eating and drinking occasion frequency was weakly associated with glucose concentrations in women and positively associated with triglyceride concentrations in both sexes; stronger associations were observed for larger vs. smaller meals and among acceptable energy reporters. Associations between meal patterns and concentration biomarkers can be observed when accounting for diurnal variation and underreporting. These findings support the use of 7-day diet diaries for studying associations between meal patterns and health.

## 1. Introduction

How a person’s diet is dispersed over the day is called “eating pattern” or “meal pattern”. It is characterized by the size, timing, frequency and skipping eating occasions [1]. Small-scale trials and observational studies show heterogeneous associations between eating patterns and cardiovascular risk factors and disease risk [2,3]. These inconsistent associations may be because of differences in study design, study duration and the various ways researchers have defined and quantified eating patterns [1,4]. Validation of eating patterns has often been limited to long-term measures such as anthropometry [5,6]; however, short-term biomarkers of intake in large observational studies could (i) validate the observed eating patterns beyond description of the self-reported diet, (ii) shed light on post-prandial responses and (iii) may describe the role of systematic reporting bias when studying associations between eating patterns and markers of disease risk outcomes.

Non-fasting triglyceride and glucose concentrations could be explored not just as a post-prandial cardiovascular disease risk factors [7,8], but also as biomarkers of eating patterns. They are metabolically interrelated and responsive to food intake [9]. Triglycerides are present in plasma in the form of post-prandial chylomicrons (in response to dietary fat) and in very low density lipoproteins from de novo lipogenesis in the liver (in response to glucose/fructose) [10]. Triglyceride concentrations rise as early as 10 min after a meal and peak at 3–4 h, gradually increasing over the day due to consecutive meals [11] with inconclusive evidence for circadian variation [12]. Circulating glucose results from digestion of simple and complex carbohydrates; alternatively, from glycolysis or gluconeogenesis when in fasting state. Glucose concentrations follow a circadian rhythm, due to insulin sensitivity declining in the afternoon resulting in higher glucose concentrations, known as ‘afternoon diabetes’ [13,14].

An understudied element in observational studies is the diurnal variation which exists in the biomarker or risk factor collected; it is often the mean value which is used [15]. Exploring diurnal variation may clarify potential associations between eating patterns and health while considering other factors which may bias this association such as dietary underreporting (e.g., the inverse association observed between meal frequency and body weight) [16]. Studies using doubly-labelled water, have shown that energy intake in diet diaries may be underreported by 17–23% of the study participants, who underreport ~20% of their daily energy expenditure [17]. Doubly-labelled water however cannot be used to validate energy distribution over the day; moreover, time of day variation in food omissions could potentially bias associations between eating patterns and health [18].

We aimed to validate observed eating patterns in a population-based cohort by studying associations between skipping, timing, size (i.e., energy content) and frequency of eating and drinking occasions (EDO) with short-term non-fasting biomarkers (glucose and triglycerides), while accounting for diurnal variation in these biomarkers and assessing heterogeneity of this association by energy reporting status.

## 2. Materials and Methods

### 2.1. Study Design

The European Prospective Investigation into Cancer based in Norfolk, UK (EPIC-Norfolk) studies the potential causes of chronic diseases [19]. Men and women between 39 and 79 years were recruited between 1993 and 1998 from 35 general practitioners’ practices. They received a general health and lifestyle questionnaire, attended a health examination where a nurse took measures of anthropometry and blood samples. Participants completed a variety of dietary assessment instruments around the time of the health examination [20]. Ethical approval was obtained from the Norwich District Health Authority Ethics Committee. Participants provided written informed consent (*N* = 25,636).

### 2.2. Eating Pattern Assessment

Diet was reported using a 7-day diet diary (7dDD), with a 99% response rate. The days were pre-structured into eight recording sections: before breakfast, breakfast, mid-morning, lunch, tea, dinner, later in the evening and a food checklist with unknown time of consumption. Participants received instructions in the form of an interviewed 24 h diet recall by a trained nurse during their health examination visit; the remaining 6 days were completed by the participant at home. Written instructions and color photographs in the 7dDD helped with portion size estimation and accurate food description. Diaries were entered, checked and calculated by trained staff into Data Into Nutrients for Epidemiological Research [21] and the program DINERMO [22]. The underlying food composition tables were McCance and Widdowson’s The Composition of Foods, 5th edition, 6th edition and the supplements. For each recording section, energy content of reported foods was summed (referred to as reported energy intake [rEI]); the mean rEI over the number of recording days represented “meal size” (MJ/d), which was also expressed as a percentage of mean daily energy intake (DEI%). Based on results from reviews [2,4], we considered any recording section which exceeded 210 kJ (50 kcal) to be an EDO. Where the recording section was <210 kJ, the recording section was considered as ‘skipped’. Where the recording section was >210 kJ but contained <15% of the DEI, it was labelled ‘EDO < 15% DEI’ (‘snack’). Recording sections containing >15% of DEI were labelled ‘EDO > 15% DEI’ (‘meal’). We calculated mean frequencies of reported daily/total EDO, EDO > 15% DEI and EDO < 15%DEI.

### 2.3. Diurnal Variation in Non-Fasting Biomarkers

Participants attended the research facility in/near their general practitioner’s practice for a health examination. Appointments were offered between 8:00 and 17:00 (later on request) and participants could indicate whether they preferred a morning or afternoon appointment. The hour at which the blood sample was taken varied between 08:00 and 19:00. The hours 08:00 and 09:00 were combined to 09:00 and 18:00 and 19:00 were combined to 18:00, to ensure reasonable sized groups, resulting in ten one-hour categories. Participants were asked at which time they last ate/drank, from which the hours fasted were calculated (minutes). Non-fasting venous blood samples were taken [19]. Serum glucose was assessed using Olympus AU640 with a detection range between 0.6 and 45.0 mmoL/L. Serum triglyceride concentrations (mmoL/L) were measured using a Technicon analyser (Bayer Diagnostics, Basingstoke, UK).

### 2.4. Adjustment Variables

Socio-demographic variables. The baseline health and lifestyle questionnaire asked about marital status. The five answer categories were regrouped into two categories: married and not married (widowed, single, separated and divorced). The highest education obtained was grouped into no qualification/less than O-level, O-level (up to the age of ~16 years), A-level (up to the age of ~18 years), degree or equivalent. Social class was measured as occupational status and grouped into manual (professional, managerial, skilled non-manual) and non-manual (skilled manual, semi-skilled, non-skilled) [23].

Lifestyle variables. Physical activity was self-reported using a questionnaire. It consisted of two components, physical activity at work and leisure time physical activity. Participants were classified into one of four groups: inactive, moderately inactive, moderately active and active. This scale has good repeatability (weighted kappa = 0.6) and has been validated against objectively measured cardio-metabolic measures, observing positive associations [24]. Smoking status was obtained from two questions on the baseline health and lifestyle questionnaire: “Do you smoke now?” and “Have you smoked as much as 1 cigarette/day for a year?”. The answers to these questions were combined into three mutually exclusive groups: never, former and current smokers.

Anthropometric variables. Participant’s height (cm) was measured without shoes using a free-standing stadiometer. Values were rounded to the nearest millimetre. Participant’s weight was measured with the participant wearing light clothing, without shoes, using a digital scale (Salter). Weight was rounded to the nearest 0.2 kg. BMI was calculated by dividing weight by the square of height in meters (kg/m^2^). The estimated Basal Metabolic Rate (BMR, MJ/d) was calculated using the Henry equations [25], making use of the participant’s height, weight, sex and age. Waist circumference was ascertained at end of expiration using non-stretch tape, placed at the minimum circumference position; and if not present, around the navel (cm).

### 2.5. Stratification/Subgroup Variables

All analyses were stratified by sex for three reasons, (i) energy needs of men are approximately 2 MJ/d higher than in women [25], potentially resulting in a different eating pattern, (ii) underreporting is more common in women than men and may affect different foods [26,27], (iii) visceral fat distribution is different in men and women, which affects insulin resistance [28]. In subgroups analysis, participants were categorized by reporting status into ‘low energy reporters’ (LER) and ‘acceptable energy reporters’ (AER). For this we made use of the assumptions by Black [29] and the 25th (1.49), 50th (1.63) and 75th (1.78) centile of physical activity level taken from the SACN report [25]. We matched these with the self-reported questionnaire physical activity described above (inactive [1.49], moderately inactive/active [1.63] and active [1.78]). Cut-offs between LER and AER and between AER and high energy reporters by physical activity were: inactive 1.02/2.18, moderately (in)active 1.12/2.38 and active 1.22/2.60.

### 2.6. Participant Selection

Participants who attended the health check (*n* = 25,636), provided a blood sample in which triglyceride concentrations were assessed (*n* = 23,866) and returned their 7dDD (*n* = 25,501) were eligible for analysis, leaving 23,752 participants. We excluded participants who were taking lipid-lowering medication (*n* = 352) or medication to control blood glucose (*n* = 437) and who completed a 7dDD reporting nightshifts or days of ill health (*n* = 395). We further excluded participants who had missing data for one or more of the adjustment variables: marital status (*n* = 128), education (*n* = 14), social class (*n* = 483), physical activity (*n* = 1), smoking (*n* = 192), BMI (*n* = 37), waist (*n* = 22), hour of blood sample (*n* = 290) and hours fasted (n = 498), leaving 21,318 participants for analysis. Data analyses of serum glucose included 16,516 participants since this biomarker was only available on a subset.

### 2.7. Statistical Analysis

Participants’ characteristics are described by hour of blood sampling. The above-described characteristics were selected based on potential associations with rEI and the hour at which the blood sample was collected or the biomarker concentration itself. Diurnal variation of the biomarkers is graphed; a description of the interrelating aspects of the eating patterns is given (EDO size i.e., rEI, EDO frequency and EDO timing).

Triglyceride and glucose concentrations were explored as potential concentration biomarkers of EDO size, EDO skipping and EDO timing. Triglyceride and glucose concentrations were log-transformed to normalize residuals. Log-transformation also helps to compare the biomarkers, since the betas represent a percent increment in the concentration. Biomarkers were adjusted for: age, socio-demographic (social class, education, marital status), lifestyle (smoking, physical activity), anthropometry (waist circumference, body mass index), season, DEI (MJ/d), frequency of EDO < 15%DEI and EDO > 15%DEI, hour of blood sample collection and EDO size. We *a priori* used interaction terms in ANCOVA models to assess whether higher rEI at an EDO (breakfast, lunch, dinner) would be associated with higher biomarker concentrations taken from blood samples in those hours directly following the respective EDO (referred to as “time-mealsize-dependent associations”). The back-transformed estimated marginal means of the biomarkers by hour of blood sampling were graphed for four percentiles of rEI: 0 MJ/d (i.e., skipped), and at the 25th, 50th and 75th centile. The difference in biomarker concentration between the 75th and the 25th centile of rEI were graphed on the *Z*-axis to quantify the observed interaction. Significant associations with interactions are visible as non-parallel lines at certain times of the day, indicating responsiveness to the rEI of a specific EDO. We tested observed associations by subgroups of reporting status (AER vs. LER). To aid comparisons between the various subgroups and the main analysis, we retained the interaction term (meal size*sample time) in any subgroup analysis if such an interaction was observed in the main analysis.

In assessing associations between EDO frequency and biomarkers, we present unadjusted (model 0), age and waist (model 1) and maximally adjusted (model 2) models. We explored variations of additive (i.e., model resulted in higher DEI or EDO frequency) and substitution models (i.e., model held DEI constant but shifted the rEI differently over the day). The statistical analyses were undertaken using IBM SPSS v25.

## 3. Results

### 3.1. Participant Characteristics

Participants were, on average, 59 years old and 54% were women (Tables S1 and S2). Men and women arriving for their health examination between 10:00 and 15:00 were on average older than those arriving earlier or later in the day. Those who attended late afternoon appointments had a larger dinner, whereas morning appointments were associated with a larger lunch. DEI, frequency of EDO > 15%DEI and EDO < 15%DEI were not strongly associated with appointment time. Early morning appointments were associated with more inactivity and late afternoon appointments with the lowest percentage of former smokers. Women arriving after 17:00, were more likely to have a higher education degree or equivalent, whereas in men, a manual occupation was more common. 97% of the participants fasted for less than 8 h. For blood samples taken between 11:00 and 12:00 and 16:00 and 18/19:00, participants had been fasting for the longest amount of time, approximately 190 min. The shortest fasting time was at the earliest appointments and at 14:00, approximately 130–150 min.

### 3.2. Diurnal Variation in Non-Fasting Triglyceride and Glucose Concentrations

**Figure 1:**
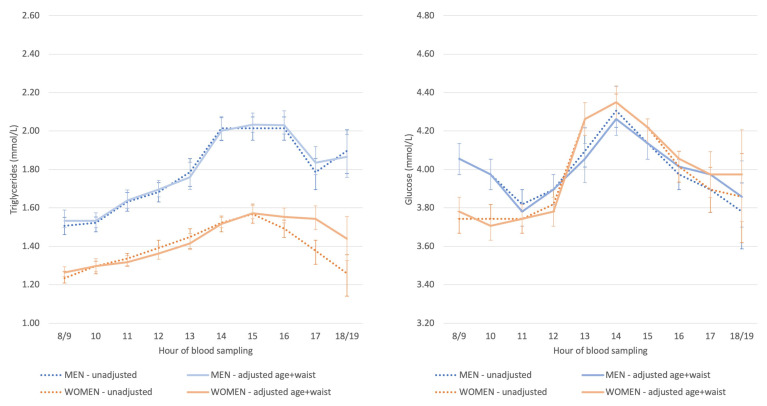
Estimated marginal means (95%CI) of triglyceride and glucose concentrations stratified by sex and adjusted for age and waist circumference (continuous). The axes have been made comparable with the axes in subsequent figures to facilitate comparisons.

Triglyceride concentrations were higher among men than women, but with a similar hour-to-hour pattern (Figure 1). Glucose concentrations in the morning were higher in men than women, whereas in the early afternoon, this pattern was reversed. The highest glucose concentration occurred in the early afternoon in both sexes. Adjustment for age and waist circumference left these associations largely unaltered.

### 3.3. Meal Pattern Description and Correlations between Size, Frequency and Timing of EDO

**Figure 2:**
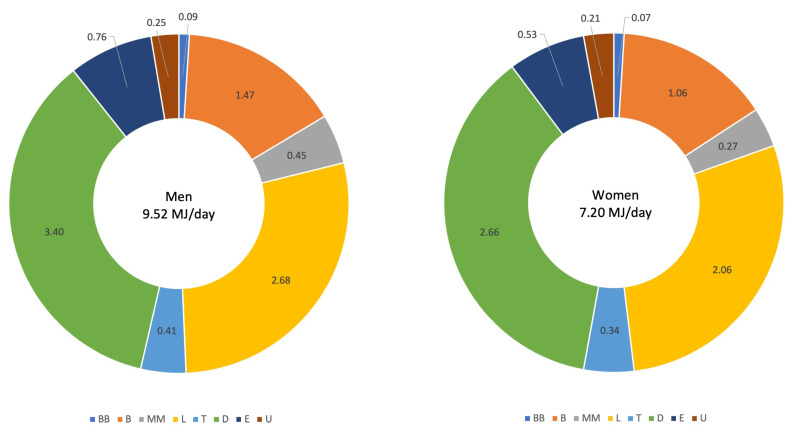
Mean reported energy intake distribution over the day (MJ/d). Data are unadjusted, grouped by sex. Abbreviations: BB, before breakfast; B, breakfast; MM, mid-morning; L, lunch; T, tea; D, dinner; E, evening; U, unknown.

Despite that DEI was 2 MJ/d higher among men compared to women, the dispersion of DEI over the day and EDO frequency were similar, with the majority of rEI eaten at evening meal, lunch and breakfast (Figure 2).

**Figure 3:**
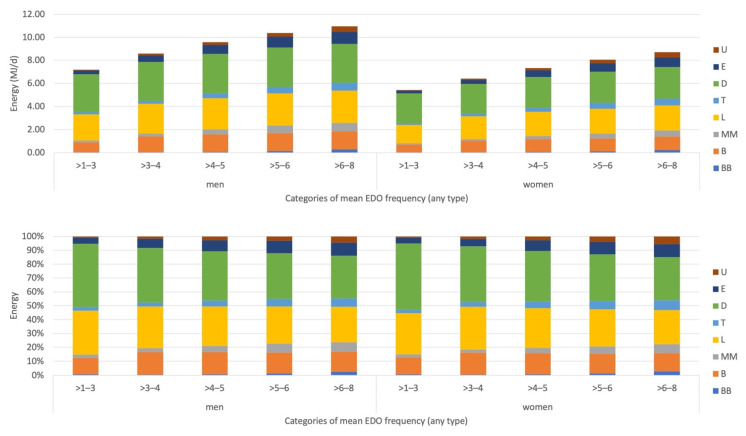
Mean reported energy intake for every recording section by EDO frequency, results are presented in absolute DEI (**top**, MJ/d) and relative to DEI (**bottom**, %DEI).

**Figure A1:**
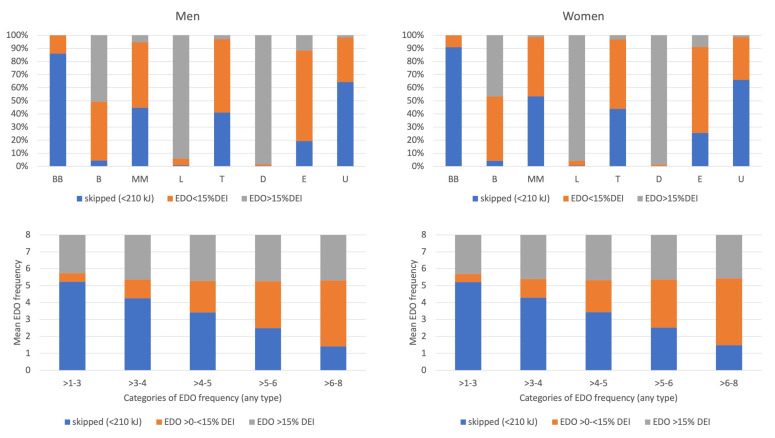
EDO Size and EDO Skipping by Recording Section (top) and by EDO Frequency (bottom). Abbreviations: EDO, Eating and Drinking Occasion; BB, before breakfast; B, breakfast; MM, mid-morning; L, lunch; T, tea; D, dinner; E, evening; U, unknown.

The recording sections before breakfast, mid-morning, tea and evening were more likely to be skipped (Figure A1, Appendix A). Regardless of the number of EDO per day, the mean number of EDO > 15%DEI were approximately similar (range 2.3–2.7/day). Therefore, a higher EDO frequency was associated with a higher number of EDO < 15%DEI (range 0.5–3.9/day), resulting in higher rEI between meals (mid-morning, tea and/or evening recording section) as well as a higher DEI (Figure 3). This pattern can also be observed in Table A1, Appendix B, where DEI was more strongly related to mid-morning, tea and/or evening recording sections. When studying the reciprocal associations between the various recording sections, we observed the strongest, inverse correlations between rEI at dinner and lunch (*r* = −0.39, −0.47), dinner and tea (*r* = −0.28, −0.29) and dinner and breakfast (*r* = −0.25, −0.25) in men and women respectively. In other words, participants consuming a larger dinner were more likely to have a smaller lunch, for example.

### 3.4. Associations between Eating Patterns and Triglyceride Concentrations

After adjustment, associations between EDO frequency and triglyceride concentrations were stronger than between DEI and this biomarker (Table 1). Minimal adjustment (model 1) compared to maximum adjustment (model 2) did not change the associations meaningfully. After mutual adjustment for DEI and EDO, every EDO more was associated with 2–3% higher triglyceride concentration. When changing the dispersion of the same amount of DEI over the day, eating one EDO > 15%DEI more was associated with a 5% higher concentration of triglycerides. Adding one EDO < 15% at the cost of an EDO > 15%, made associations with triglycerides negative, whereas, adding one EDO < 15% extra to the day, was associated with higher triglyceride concentrations. After including both EDO types, effect sizes of EDO > 15%DEI were approximately 3-fold stronger compared to EDO < 15%DEI, both without (d) and with (e) DEI adjustment.

**Figure 4:**
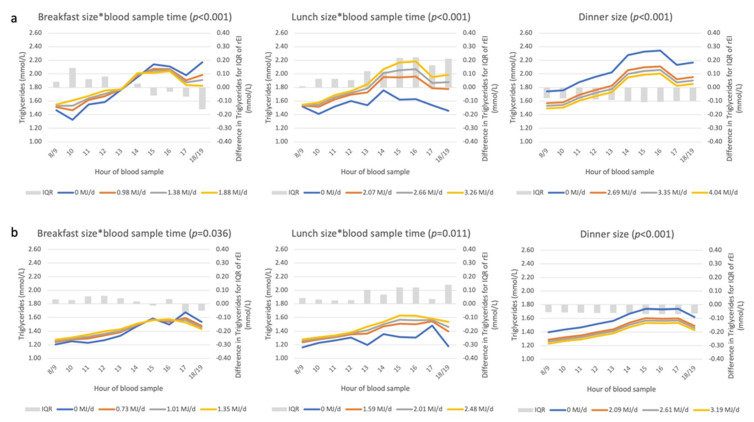
Associations between rEI at three recording sections (“meal size”) and triglyceride concentrations among men (**a**) and women (**b**). Abbreviations: DEI, daily energy intake (MJ/d); IQR, interquartile range; rEI, reported energy intake. The *p*-value represents the statistical significance of the interaction term between rEI and time (*), as assessed with the F-test. Where the interaction term was non-significant, the *p*-value is the statistical significance of the recording section size, as assessed with the F-test. See Appendix D for description of axes, model and interpretation of the graph.

We then studied whether the association between blood sampling time and triglycerides was modified by rEI at main meals to assess face validity of the meal pattern (Figure 4). Time–meal size-dependent associations with breakfast and lunch size were observed, i.e., those reporting a larger breakfast or lunch had a higher triglyceride concentration only in the hours following these respective EDOs. Skipping breakfast was associated with lower triglyceride concentrations at the earliest appointments, stronger in men. Those reporting to skip lunch, had consistently lower triglyceride concentrations in the afternoon compared to those consuming lunch. The largest dinner size was associated with the lowest triglyceride concentrations during the day, regardless of the appointment time (i.e., no interaction).

### 3.5. Associations between Eating Patterns and Glucose Concentrations

In men, we observed weaker and fewer associations between EDO frequency and glucose concentration compared to women (Table 2). In women, every additional EDO > 15%DEI (in additive and substitution models) was associated with 2% higher glucose concentration; no association was observed for EDO < 15%DEI, except when an EDO > 15%DEI was replaced with an additional EDO < 15%DEI, reversing the association.

**Figure 5:**
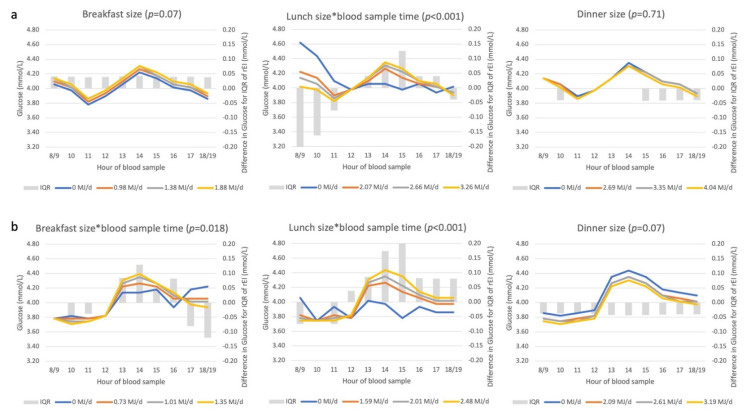
Associations between rEI at three recording sections (‘meal size’) and glucose concentrations among men (**a**) and women (**b**). Abbreviations: DEI, daily energy intake (MJ/d); IQR, interquartile range; rEI, reported energy intake. The *p*-value represents the statistical significance of the interaction term between rEI and time (*), as assessed with the F-test. Where the interaction term was non-significant, the *p*-value is the statistical significance of the recording section size, as assessed with the F-test. See Appendix D for description of axes, model and interpretation of the graph.

The association between blood sampling time and glucose was modified by meal size, but mainly for lunch (Figure 5). In men, no association was observed between breakfast and glucose concentrations throughout the day. Whereas among women, regardless of breakfast size, glucose remained approximately constant in the morning, but higher rEI at breakfast was associated with higher glucose concentrations in the early afternoon. In both sexes, glucose concentrations were higher in the afternoon compared to the morning; moreover, a time–meal size-dependent association was observed between rEI at lunch and glucose concentrations (*p* < 0.001). Those skipping lunch, had the lowest glucose concentrations in the afternoon and higher rEI at lunch was associated with higher glucose concentrations in the afternoon. Lunch skippers also had the highest glucose concentrations at early morning appointments, particularly among men. No associations were observed between rEI at dinner and glucose concentrations during the day.

### 3.6. Underreporting Modifies Associations between Biomarkers and Eating Patterns

We repeated the association between EDO frequency and biomarker concentrations by energy reporting status. Of the men, 80% were AER compared to 75% of the women; characteristics of AER and LER are reported in Table S3.

Only among AER did we observe that a higher EDO < 15%DEI frequency was positively associated with triglyceride concentrations (Table 1). Among AER, associations between EDO frequency and glucose were stronger compared to the full sample, but associations remained weak overall (Table 2).

**Figure 6:**
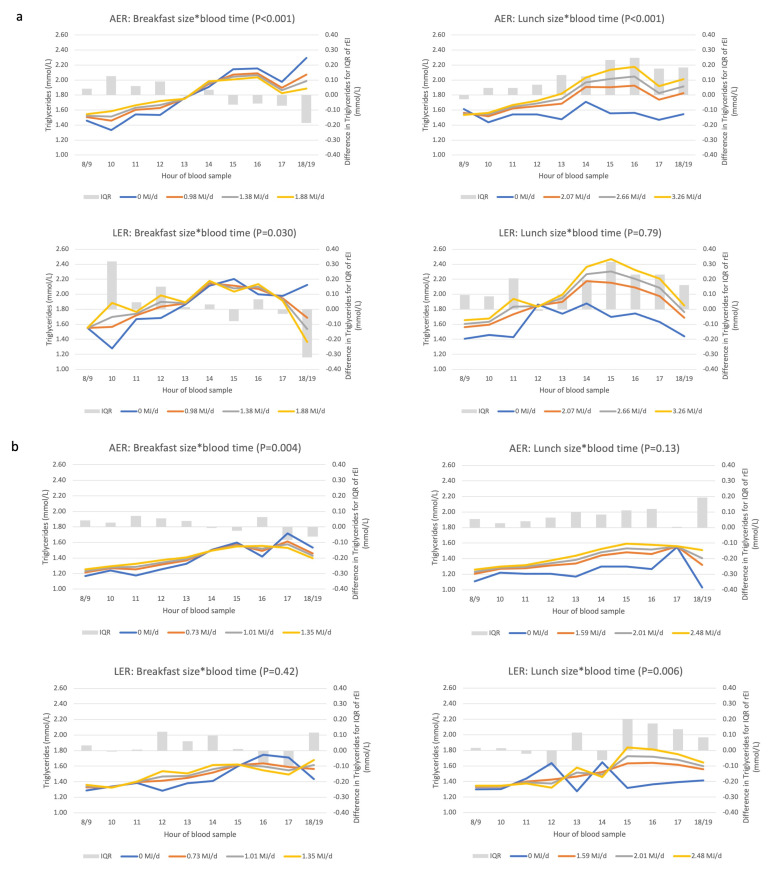
Associations between rEI (‘meal size’) and triglyceride concentrations by reporting status in men (**a**) and women (**b**). Abbreviations: AER, acceptable energy reporter; DEI, daily energy intake (MJ/d); IQR, interquartile range; LER, low energy reporter; rEI, reported energy intake. See Appendix D for a description of the axes and Table S3 for a description of LER and AER.

**Figure 7:**
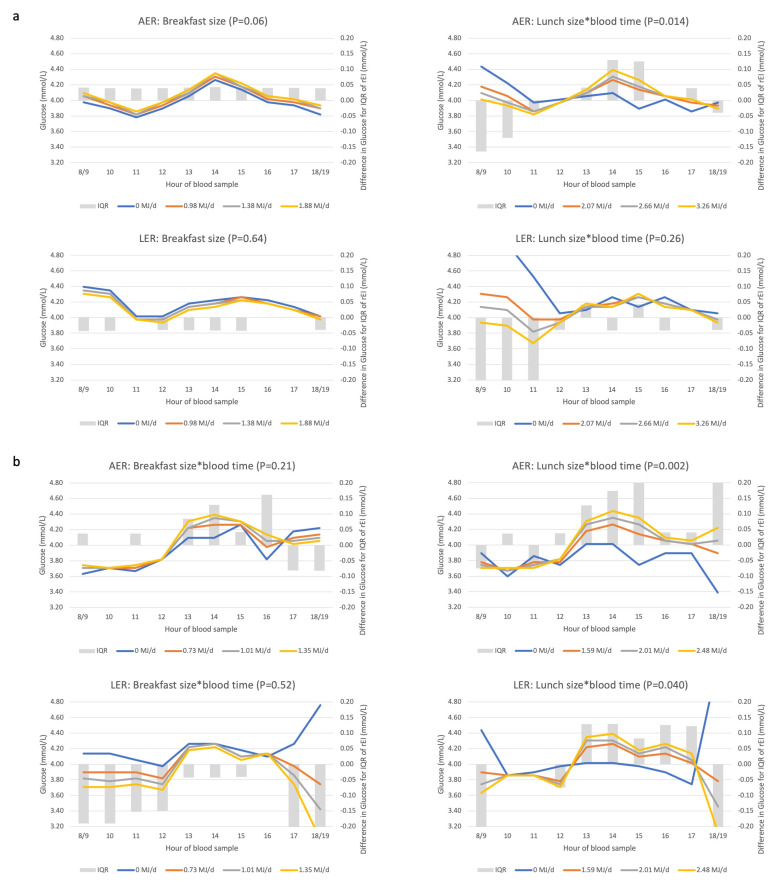
Associations between rEI (‘meal size’) and glucose concentrations by reporting status in men (**a**) and women (**b**). Abbreviations: AER, acceptable energy reporter; DEI, daily energy intake (MJ/d); IQR, interquartile range; LER, low energy reporter; rEI, reported energy intake. See Appendix D for a description of the axes and Table S3 for a description of LER and AER.

We also repeated the time–meal size biomarker association by reporting status. Among men and women who were LER compared to AER, the same rEI was associated with more variable triglyceride concentrations (Figure 6); whereas skipping breakfast or lunch was associated with the lowest triglyceride concentrations in both LER and AER. With regards to glucose and among AER only, higher rEI at lunch was associated with higher glucose concentrations in the afternoon (Figure 7); whereas among LER, glucose remained higher for a longer period of time during the afternoon.

## 4. Discussion

### 4.1. Statement of Principal Findings

For both biomarkers and diet, we observed diurnal variation. We used this variation to assess face validity of the reported meal patterns (meal size, meal timing and meal frequency). Positive time–meal-size-dependent associations could be observed between rEI at breakfast and triglyceride concentrations in the morning and between rEI at lunch and triglyceride as well as glucose concentrations in the afternoon. Those skipping breakfast had the lowest triglyceride concentrations in the morning and those skipping lunch had the lowest triglyceride and glucose concentrations in the afternoon, more clearly among AER. EDO frequency was weakly associated with glucose concentrations. Frequency of EDO > 15%DEI was three times more strongly associated with triglyceride concentrations than EDO < 15%DEI. Associations between EDO < 15%DEI and triglycerides were only observed among AER.

### 4.2. Strengths and Limitations of This Study

Strengths of this study are the large sample size of this population-based cohort of both men and women, mostly in post-prandial state. Dietary data were collected using detailed open-ended questionnaires, enabling a time–meal-size-dependent analysis with biochemical indicators of intake. Anthropometry was measured and these data were subsequently used to assess the extent of underreporting and its influence on the observed associations between eating patterns and biomarkers.

Limitations include that the 7dDD had pre-structured recording sections. These provided a relative order, but not an exact time, resulting in potential (random) mismatch between the blood sample time and eating time, attenuating associations. Foods recorded in the same recording section were counted as a single EDO which is likely to have underestimated the number of daily EDO, especially EDO < 15%DEI. Fewer late afternoon appointments were scheduled; therefore, associations had larger confidence intervals at these times, limiting the power in interaction analysis. Our biomarker data covered the hours from 08:00 till 19:00, and we were therefore unable to ascertain validity of eating late at night, which has been associated with cardiovascular disease risk [30]. Glycaemic index/load could have influenced post-prandial response which we could not adjust for because of unavailability of this variable. Although we adjusted for a wide range of variables associated with DEI and time of blood sampling, we cannot exclude unmeasured and residual confounding.

### 4.3. Results in Context of Other Studies

We have described meal size, frequency and timing of EDO and observed that meal size (rEI) varies by time of day and frequency of EDO. Such observations have been described for countries across the globe [31,32,33,34,35]. Methods for identifying meal patterns vary from study to study, and are likely to lead to differences in observed patterns, especially EDO frequency [1,2]. Here, we have presented the validation results using EDO definitions as recommended in a recent review [2] and observed that higher frequency of EDO > 15%DEI was associated with greater magnitude in higher triglyceride concentrations compared to higher frequency of EDO < 15%DEI, indicating that a distinction between self-reported meal sizes in this observational study can be made. However, associations regarding EDO frequency were stronger among AER compared to LER and only among AER could associations between EDO < 15%DEI frequency and triglycerides be observed. This finding may be explained by underreporting of snacks in LER [16]. Associations between EDO frequency and glucose concentrations were weaker compared to triglycerides. Differences between these biomarkers may reflect stronger counter-regulatory pathways in glucose control [36]. A higher DEI at the same EDO frequency was not associated with higher biomarker concentrations, indicating that meal frequency was more important for post-prandial concentrations. Breakfast and lunch recording sections in the 7dDD without any recorded foods, were associated with the lowest concentrations of glucose and triglycerides, providing some evidence that these indeed reflect skipped meals, rather than under recorded meals. The biomarker curves varied at which time points the curves for the 25th, 50th and 75th centile of rEI separated, which can be considered an indication of meal size. The curves cannot represent the effect sizes we see with, for example, continuous glucose monitoring, but in this observational setting we observe that larger breakfast and lunch are associated with higher biomarker concentrations, giving face validity to the 7dDD assessment. Time–meal-size-dependent associations were only observed for the recording sections traditionally labelled as “main meals” and not for the recording sections in between. This is most likely explained by lower rEI at non-main meals; alternatively, eating between meals may not have resulted in (even) higher concentrations but a pro-longed post-prandial state since post-prandial clearance of triglycerides takes several hours [11], also hourly measures of glucose in high compared to low frequency trial diets appear stable and high during the day [37]. Our observation that higher rEI at dinner was associated with lower triglyceride concentrations during the day is likely non-causal and the result of reciprocal associations between recording sections, i.e., participants who report large dinners were more likely to report smaller main meals during the day (Table A1).

In men, compared to women, we observed higher mean triglyceride concentrations at every time point and a greater magnitude in the time–meal-size-dependent association. Such differences may be attributed to abdominal compared to gluteo-femoral adiposity [38], where the latter has been associated with better insulin sensitivity and lipid profile. Additionally, triglyceride and glucose clearance may have been worse among those with larger waists due to insulin resistance and less available lipoprotein lipase [39,40]; moreover, glycerol from visceral fat may have been a contributor to circulating glucose among those with visceral adiposity [28,41].

We aimed to remove the “flattened slope” between rEI and the biomarker by repeating the analyses among AER and LER. We did observe that time–meal-size-dependent associations were larger in magnitude among LER, which firstly needs to take into account the associated anthropometry as described above but may also be explained by the true energy intake of a meal being higher than the rEI among LER. We also observed that associations for EDO frequency among AER were stronger compared to the whole sample. However, the LER group was relatively small, and the associated anthropometric characteristics of underreporting (higher waist and BMI, Table S3) and hypertriglyceridemia made complete separation of these conditions impossible.

The proportion of energy substrates may have varied between EDO times, this could have altered nutrient metabolism [42] and the ability to respond to a high carbohydrate or fat meal followed by a meal of opposing composition (the “second meal” effect) [43]. We might have observed a stronger magnitude of associations had we used meal-specific nutrient content instead of rEI; however, glucose and fat metabolism are intertwined as are their substrates [7]. Additionally, energy intake is of specific interest in nutritional epidemiology [44] and dietary fat and carbohydrates make up the majority of the energy sources in our study (Tables S1 and S2).

Regarding external validity, participants in this cohort were found to be broadly representative of the UK population, with the exception of having a lower smoking prevalence [19]. Participants were aged 39–79 years at baseline and therefore generalization to younger and older age groups needs to take differences in meal patterns into account; moreover, glucose and triglyceride concentrations become more intolerant with aging due to changes in body weight, body mass index, waist circumference and body composition and therefore the observed time–meal-size-dependent associations may be different outside the age range included [28].

### 4.4. Relevance

Diurnal variation in dietary intake and how this relates to health is an area of interest [45,46], as is the personal response to a meal [15]. Time of consumption of meals, food groups and nutrients have been studied in relation to cardio-metabolic risk factors of disease risk [47,48,49]; however, chrono-nutrition (meal timing in relation to the body clock) also needs to take into account diurnal variation in the risk factor itself, e.g., peak glucose concentrations early in the afternoon. Participants’ preferences for a time to attend research facilities may also relate to their chronotype (morning vs. evening preference) and preferred meal pattern [50] and, therefore, we need to study further whether time of assessment in observational studies may alter the observed associations between meal time (e.g., breakfast), meal size and risk factors of disease, e.g., blood pressure [51], cholesterol [52] or body weight [53].

## 5. Conclusions

In this cohort population of private households, we observed associations between meal pattern characteristics and non-fasting glucose and triglyceride concentrations. These findings indicate that time of blood collection was an important aspect in observing diet-biomarker associations. Self-reported dietary intake can be used to study associations between meal patterns and health, once reporting status and anthropometric measures associated with insulin resistance are taken into account.

## Figures and Tables

**Table 1:** Association between DEI and/or EDO frequency and triglyceride concentrations ^a^.

Exposure	Adjustment ^b^	Log-Triglycerides (mmoL/L) Beta (95%CI)	
		Men (n 9724)	Women (n 11 594)
DEI (MJ/d)	Model 0	−0.008 (−0.012, −0.003)	−0.018 (−0.023, −0.012)
DEI (MJ/d)	Model 1	−0.002 (−0.006, 0.003)	0.004 (−0.001, 0.009)
DEI (MJ/d)	Model 2	0.001 (−0.003, 0.006)	0.007 (0.002, 0.012)
DEI (MJ/d) ^c^	Model 2 + EDO	−0.004 (−0.009, 0.001)	0.003 (−0.003, 0.009)
EDO (1 EDO/day)	Model 0	0.007 (−0.005, 0.018)	0.011 (−0.000, 0.021)
EDO (1 EDO/day)	Model 1	0.025 (0.014, 0.035)	0.021 (0.011, 0.031)
EDO (1 EDO/day)	Model 2	0.024 (0.013, 0.034)	0.020 (0.011, 0.030)
EDO (1 EDO/day) ^c^	Model 2 + DEI	0.027 (0.016, 0.039)	0.018 (0.007, 0.029)
EDO > 15%DEI	Model 2 + DEI	0.056 (0.033, 0.079)	0.050 (0.030, 0.070)
EDO > 15%DEI	Model 2 + EDO	0.050 (0.028, 0.073)	0.049 (0.029, 0.069)
EDO < 15%DEI	Model 2 + DEI	0.011 (0.000, 0.021)	0.003 (−0.007, 0.012)
EDO < 15%DEI	Model 2 + EDO	−0.050 (−0.073, −0.028)	−0.049 (−0.069, −0.029)
EDO > 15%DEI ^d^	Model 2 + EDO < 15%DEI	0.071 (0.047, 0.095)	0.067 (0.046, 0.088)
EDO < 15%DEI ^d^	Model 2 + EDO > 15%DEI	0.021 (0.010, 0.031)	0.018 (0.009, 0.028)
EDO > 15%DEI ^e^	Model 2 + EDO < 15%DEI + DEI	0.078 (0.054, 0.103)	0.064 (0.042, 0.086)
EDO < 15%DEI ^e^	Model 2 + EDO > 15%DEI + DEI	0.026 (0.014, 0.037)	0.016 (0.005, 0.027)
***Among AER***			
EDO > 15%DEI ^f^	Model 2 + EDO < 15%DEI + DEI	0.078 (0.050, 0.106)	0.071 (0.045, 0.096)
EDO < 15%DEI ^f^	Model 2 + EDO > 15%DEI + DEI	0.024 (0.011, 0.036)	0.020 (0.008, 0.032)
***Among LER***			
EDO > 15%DEI ^g^	Model 2 + EDO < 15%DEI + DEI	0.075 (0.022, 0.127)	0.050 (0.008, 0.093)
EDO < 15%DEI ^g^	Model 2 + EDO > 15%DEI + DEI	0.027 (−0.002, 0.056)	0.002 (−0.023, 0.027)

^a^ Abbreviations: DEI, daily energy intake (MJ/d); EDO, eating and drinking occasion (any type); EDO > 15%DEI, EDO containing more than 15%DEI; EDO < 15%DEI, EDO containing less than 15%DEI; AER, acceptable energy reporter; LER, low energy reporter. ^b^ ANCOVA Model 0: unadjusted. Model 1: Age and waist circumference. Model 2: Age, waist circumference, marital status, social class, education, smoking, physical activity, BMI, hours fasted, season, hour of blood sample. ^c–g^ Results have been derived from a statistical model with mutual adjustment. The biomarkers were log-transformed, results may be interpreted as % change, e.g., exp [0.024] = 1.024 = +2.4%. The coefficients may be interpreted as described in Appendix C: Interpretation of the Association between DEI and/or EDO Frequency and Biomarker Concentrations.

**Table 2:** Association between DEI and/or EDO frequency and glucose concentrations ^a^.

Exposure	Adjustment ^b^	Log-Glucose (mmoL/L) Beta (95%CI)	
		Men (n 7395)	Women (n 9121)
DEI (MJ/d)	Model 0	−0.002 (−0.005, 0.001)	−0.002 (−0.005, 0.002)
DEI (MJ/d)	Model 1	0.001 (−0.002, 0.005)	0.003 (−0.001, 0.007)
DEI (MJ/d)	Model 2	0.002 (−0.002, 0.005)	0.004 (0.000, 0.007)
DEI (MJ/d) ^c^	Model 2 + EDO	0.003 (−0.000, 0.007)	0.004 (−0.000, 0.008)
EDO (1 EDO/day)	Model 0	−0.007 (−0.014, 0.001)	0.004 (−0.003, 0.011)
EDO (1 EDO/day)	Model 1	−0.006 (−0.013, 0.002)	0.005 (−0.002, 0.011)
EDO (1 EDO/day)	Model 2	−0.006 (−0.014, 0.001)	0.003 (−0.004, 0.009)
EDO (1 EDO/day) ^c^	Model 2 + DEI	−0.009 (−0.018, −0.001)	−0.001 (−0.009, 0.007)
EDO > 15%DEI	Model 2 + DEI	−0.002 (−0.019, 0.014)	0.022 (0.007, 0.036)
EDO > 15%DEI	Model 2 + EDO	0.000 (−0.016, 0.017)	0.022 (0.008, 0.037)
EDO > 15%DEI ^d^	Model 2 + EDO < 15%DEI	−0.006 (−0.023, 0.011)	0.024 (0.008, 0.039)
EDO < 15%DEI	Model 2 + DEI	−0.007 (−0.015, 0.000)	−0.006 (−0.013, 0.001)
EDO < 15%DEI	Model 2 + EDO	0.000 (−0.017, 0.016)	−0.022 (−0.037, −0.008)
EDO < 15%DEI ^d^	Model 2 + EDO > 15%DEI	−0.006 (−0.014, 0.001)	0.001 (−0.005, 0.008)
EDO > 15%DEI ^e^	Model 2 + EDO < 15%DEI + DEI	−0.011 (−0.028, 0.007)	0.020 (0.004, 0.036)
EDO < 15%DEI ^e^	Model 2 + EDO > 15%DEI + DEI	−0.009 (−0.018, −0.001)	−0.002 (−0.009, 0.006)
***Among AER***			
EDO > 15%DEI ^f^	Model 2 + EDO < 15%DEI + DEI	−0.014 (−0.034, 0.007)	0.030 (0.012, 0.049)
EDO < 15%DEI ^f^	Model 2 + EDO > 15%DEI + DEI	−0.012 (−0.021, −0.003)	0.003 (−0.006, 0.011)
***Among LER***			
EDO > 15%DEI ^g^	Model 2 + EDO < 15%DEI + DEI	−0.003 (−0.040, 0.035)	−0.003 (−0.033, 0.028)
EDO < 15%DEI ^g^	Model 2 + EDO > 15%DEI + DEI	0.001 (−0.020, 0.021)	−0.015 (−0.033, 0.003)

^a^ Abbreviations: DEI, daily energy intake (MJ/d); EDO, eating and drinking occasion (any type); EDO > 15%DEI, EDO containing more than 15%DEI; EDO < 15%DEI, EDO containing less than 15%DEI; AER, acceptable energy reporter; LER, low energy reporter. ^b^ ANCOVA Model 0: unadjusted. Model 1: Age and waist circumference. Model 2: Age, waist circumference, marital status, social class, education, smoking, physical activity, BMI, hours fasted, season, hour of blood sample. ^c–g^ Results have been derived from a statistical model with mutual adjustment. The biomarkers were log-transformed, results may be interpreted as % change, e.g., exp [0.024] = 1.024 = +2.4%. The coefficients may be interpreted as described in Appendix C.

## Data Availability

Data and results described in the manuscript will not be made available. The EPIC-Norfolk study is however open to collaboration. Please contact the EPIC-Norfolk management team to register and approve your data request.

## Supplementary Materials

The following are available online at: https://www.mdpi.com/article/10.3390/nu14020238/s1. Table S1: Description of EPIC-Norfolk participants (MEN only) by hour at which their blood sample was taken at the first health examination (1993–1998). Table S2: Description of EPIC-Norfolk participants (WOMEN only) by hour at which their blood sample was taken at the first health examination (1993–1998). Table S3: Characteristics of EPIC-Norfolk participants by energy reporting status.

## Abbreviations

7dDD, 7-day diet diary; AER, acceptable energy reporter; DEI, daily energy intake; EPIC-Norfolk, European Prospective Investigation into Cancer based in Norfolk (United Kingdom); EDO, eating and drinking occasion, a recording section exceeding 120 kJ/50 kcal; EDO > 15%DEI, eating and drinking occasion which contains *more* than 15% of the daily energy intake (‘meal’), independent of the time of consumption; EDO < 15%DEI, eating and drinking occasion which contains *less* than 15% of the daily energy intake (‘snack’), independent of the time of consumption; LER, low energy reporter (under reporter); Recording sections, the pre-structured day sections in the paper version of the 7dDD: before breakfast (BB), breakfast (B), mid-morning (MM), lunch (L), tea (T), dinner (D), evening (E), unknown (U). Any of these sections can be either type of EDO (or skipped) depending on the reported energy intake; rEI, reported energy intake.

## Appendix B. Correlations between DEI and the Percentages of DEI Consumed at the Eight Recording Sections in the Pre-Structured 7dDD

**Table A1:** Correlations between DEI and the Percentages of DEI Consumed at the Eight Recording Sections in the Pre-Structured 7dDD.

		DEI (MJ/d)	BB (%DEI)	B (%DEI)	MM (%DEI)	L (%DEI)	T (%DEI)	D (%DEI)	E (%DEI)	U (%DEI)
	men	9.52 (2.19)	1.0 (1.8)	15.4 (7.3)	4.5 (5.2)	28.0 (8.9)	4.2 (4.5)	36.0 (9.8)	7.8 (6.4)	2.6 (4.0)
	women									
**DEI (MJ/d)**	7.20 (1.63)		−0.004	−0.016	0.161 **	−0.096 **	0.059 **	−0.110 **	0.129 **	0.033 **
**BB (%DEI)**	1.0 (1.6)	−0.009		−0.088 **	0.022 *	−0.102 **	0.080 **	−0.089 **	0.022 *	0.016
**B (%DEI)**	14.8 (6.4)	0.007	−0.090 **		−0.239 **	−0.121 **	−0.091 **	−0.253 **	−0.237 **	−0.108 **
**MM (%DEI)**	3.7 (3.6)	0.125 **	0.056 **	−0.187 **		−0.235 **	0.106 **	−0.181 **	0.002	−0.035 **
**L (%DEI)**	28.7 (8.4)	−0.114 **	−0.096 **	−0.101 **	−0.202 **		−0.187 **	−0.394 **	−0.223 **	−0.127 **
**T (%DEI)**	4.7 (4.4)	0.116 **	0.054 **	−0.117 **	0.140 **	−0.180 **		−0.276 **	−0.020 *	−0.011
**D (%DEI)**	37.1 (9.6)	−0.097 **	−0.099 **	−0.253 **	−0.167 **	−0.465 **	−0.287 **		−0.240 **	−0.149 **
**E (%DEI)**	7.2 (5.7)	0.107 **	0.024 **	−0.221 **	0.024 **	−0.209 **	−0.007	−0.242 **		−0.057 **
**U (%DEI)**	2.8 (3.8)	0.074 **	0.030 **	−0.143 **	0.003	−0.120 **	0.004	−0.163 **	−0.069 **	

Abbreviations: DEI, daily energy intake; BB, before breakfast, B, breakfast, MM, midmorning; L, lunch; T, tea; D, dinner; E, evening; U, unknown. Results are presented cross-tabulated. Grey scale cells indicate results for women. Pearson correlation * *p* < 0.05, ** *p* < 0.01.

## Appendix C. Interpretation of the Association between DEI and/or EDO Frequency and Biomarker Concentrations

**DEI (Model 2 + EDO constant, the proportion of EDO > 15%DEI and EDO < 15%DEI may change):** For every MJ/d extra at the same EDO frequency means that EDOs (EDO > 15%DEI or EDO < 15%DEI) have to become larger (EDO < 15%DEI may turn into EDO > 15%DEI).

**EDO (Model 2 + DEI constant, re-distributing the same amount of DEI over the day):** For the number of EDOs (EDO > 15%DEI or EDO < 15%DEI) to increase at constant DEI, means that EDOs have to become smaller in order to fit more EDOs in the day.

**EDO > 15%DEI (Model 2 + DEI constant):** For the number of EDO > 15%DEI to increase at constant DEI, means that (a) the energy content of an EDO < 15%DEI becomes larger and turns into an EDO > 15%DEI while the remaining EDOs (EDO > 15%DEI or EDO < 15%DEI) become *smaller* (but retain their 15%DEI cutoff) *or* (b) that the energy content of multiple EDO < 15%DEI are replaced by a single EDO > 15%DEI (since the total EDO frequency is not held constant) *or* (c) that any of the EDOs (EDO > 15%DEI or EDO < 15%DEI) become *smaller* (but retain their 15%DEI cutoff) thereby leaving enough energy intake to create an additional EDO > 15%DEI at the same DEI.

**EDO > 15%DEI (Model 2 + EDO constant, DEI may increase):** For the number of EDO > 15%DEI to be higher at constant EDO (EDO > 15%DEI or EDO < 15%DEI), means that the energy content of an EDO < 15%DEI becomes *larger* and turns into an EDO > 15%DEI while any of the remaining EDOs retain their 15%DEI cutoff.

**EDO > 15%DEI (Model 2 + EDO < 15%DEI constant, DEI may increase):** For the number of EDO > 15%DEI to be higher while keeping the number of EDO < 15%DEI constant, means that participants are *adding* EDO > 15%DEI to their day, which may change DEI.

**EDO < 15%DEI (Model 2 + DEI constant)** For the number of EDO < 15%DEI to increase at constant DEI, means that (a) the energy content of an EDO > 15%DEI becomes smaller and turns into an EDO < 15%DEI while any of the remaining EDOs (EDO > 15%DEI or EDO < 15%DEI) become *larger* (but retain their 15%DEI cutoff) *or* (b) that any of the EDOs (EDO > 15%DEI or EDO < 15%DEI) become *smaller* (but retain their 15%DEI cutoff) thereby leaving enough energy intake to create an additional EDO < 15%DEI at the same DEI *or* (c) that the energy content of a single EDO > 15%DEI is replaced by multiple EDO < 15%DEI (since the total EDO frequency is not held constant).

**EDO < 15%DEI (Model 2 + EDO constant, DEI may increase):** For the number of EDO < 15%DEI to be higher at constant EDO (EDO > 15%DEI or EDO < 15%DEI), means that the energy content of an EDO > 15%DEI becomes *smaller* and turns into an EDO < 15%DEI while any of the remaining EDOs retain their 15%DEI cutoff.

**EDO < 15%DEI (Model 2 + EDO > 15%DEI constant, DEI may increase):** For the number of EDO < 15%DEI to be higher while keeping the number of EDO > 15%DEI constant, means that participants are *adding* EDO < 15%DEI to their day, which may change DEI.

**EDO > 15%DEI (Model 2 + EDO < 15%DEI constant, DEI constant):** For the number of EDO > 15%DEI to be higher at constant EDO < 15%DEI and constant DEI, means that any of the EDOs (EDO > 15%DEI or EDO < 15%DEI) become *smaller* (but retain their 15%DEI cutoff) thereby leaving enough energy intake to create an additional EDO > 15%DEI.

**EDO < 15%DEI (Model 2 + EDO > 15%DEI constant, DEI constant):** For the number of EDO < 15%DEI to be higher at constant EDO > 15%DEI and constant DEI, means that any of the EDOs (EDO > 15%DEI or EDO < 15%DEI) become *smaller* (but retain their 15%DEI cutoff) thereby leaving enough energy intake to create an additional EDO < 15%DEI.

## Appendix D. Description and Interpretation of the Line Graphs

The sex-specific line charts depict the associations between rEI at different recording sections (e.g., breakfast) with the biomarker, where the biomarker is the *same* variable for every recording section.

On the *X*-axis is the categorical variable of hour at which the participants attended their health examination. On the *Y*-axis, the lines represent the biomarker concentration at the 25th, 50th and 75th centile of reported energy intake (rEI). The grey bars (*Z*-axis) represent the difference in the biomarker for the IQR of rEI (75th–25th).

Lines which change in distance from each other by hour of sampling (i.e., are not parallel to each other from 08:00–19:00) indicate a different strength/direction of the association between meal size (rEI) and the biomarker (interaction).

Model adjusted for: age, marital status, social class, education, smoking, physical activity, waist circumference, BMI, hours fasted, season, DEI (MJ/d), EDO < 15%DEI frequency, EDO > 15%DEI frequency, hour of blood sample, rEI of the recording section (MJ/d), and the interaction term between hour of blood sampling and rEI of the recording section.
