# Supplementary material for: Face Validity of Observed Meal Patterns Reported with 7-Day Diet Diaries in a Large Population-Based Cohort Using Diurnal Variation in Concentration Biomarkers of Dietary Intake
Source: Nutrients. 2022 Jan 6;14(2):238. doi: 10.3390/nu14020238 (PMC8780432; doi:10.3390/nu14020238)
Supplement: Supplementary file 1 [file nutrients-14-00238-s001.zip › nutrients-1504812-Supplementary materials.pdf]

SUPPLEMENTARY MATERIALS

Face validity of observed meal patterns ascertained with 7-day diet diaries in a large population-based cohort using diurnal variation in concentration biomarkers of dietary intake

Marleen AH Lentjes, Linda M Oude Griep, Angela A Mulligan,

Scott Montgomery, Nicholas J Wareham, Kay-Tee Khaw

Supplement Table S1: Description of EPIC-Norfolk participants (MEN only) by hour at which their blood sample was taken at the first health examination (1993-1998) .. 3

Supplement Table S2: Description of EPIC-Norfolk participants (WOMEN only) by hour at which their blood sample was taken at the first health examination (1993-1998)........ 5

Supplement Table S3: Characteristics of EPIC-Norfolk participants by energy reporting status 7

Glossary

DEI, daily energy intake

7dDD, 7-day diet diary

Recording sections, the pre-structured day sections in the paper version of the 7dDD: before breakfast (BB), breakfast (B), mid-morning (MM), lunch (L), tea (T), dinner (D), evening (E), unknown (U). Any of these sections can be either type of EDO (or skipped).

rEI, reported energy intake (in a recording section).

EDO, eating and drinking occasion, a recording section exceeding 120 kJ/50 kcal.

EDO>15%DEI, eating and drinking occasion which contains *more* than 15% of the daily energy intake (‘meal’), independent of the time of consumption.

EDO<15%DEI, eating and drinking occasion which contains *less* than 15% of the daily energy intake (‘snack’), independent of the time of consumption.

AER, acceptable energy reporter

LER, low energy reporter (under reporter)

EPIC-Norfolk, European Prospective Investigation into Cancer based in Norfolk (United Kingdom)

Supplement Table S1: Description of EPIC-Norfolk participants (MEN only) by hour at which their blood sample was taken at the first health examination (1993-1998).

		8/9 (n 1122)		10 (n 1383)		11 (n 1262)		12 (n 1202)		13 (n 686)		14 (n 1090)		15 (n 1127)		16 (n 1016)		17 (n 596)		18/19 (n 240)		Men (n 9724)	
**Age (y)**		58.0	9.3	60.2	9.3	61.0	9.5	61.0	9.3	60.9	9.3	60.1	9.1	60.2	9.2	58.5	9.0	54.3	7.4	53.0	6.4	59.5	9.3
**BMI (kg/m2)**		26.4	3.1	26.5	3.3	26.4	3.3	26.3	3.1	26.6	3.5	26.6	3.3	26.5	3.2	26.4	3.3	26.2	3.4	26.8	3.4	26.5	3.3
**Waist (cm)**		95	9	96	10	96	10	95	9	97	10	96	10	95	9	95	10	94	10	96	9	96	10
**DEI (MJ/d)**		9.6	2.2	9.5	2.1	9.5	2.2	9.4	2.2	9.5	2.2	9.4	2.2	9.4	2.1	9.6	2.3	10.0	2.2	10.3	2.3	9.5	2.2
**Protein (%DEI)**		15.1	2.6	15.0	2.5	14.8	2.5	15.0	2.5	15.0	2.6	14.9	2.6	15.1	2.5	15.0	2.5	14.9	2.4	14.7	2.6	15.0	2.5
**Fat (%DEI)**		33.6	5.5	33.5	5.3	33.6	5.6	33.4	5.5	33.1	5.4	33.4	5.4	33.2	5.4	33.8	5.4	34.1	5.2	34.2	4.7	33.5	5.4
**Carbohydrate (%DEI)**		46.0	6.8	46.3	6.8	46.4	6.8	46.5	6.7	46.8	6.5	46.7	6.6	46.6	6.5	46.1	6.8	46.7	6.2	46.6	6.3	46.4	6.7
**Alcohol (%DEI)**		5.3	6.0	5.2	6.4	5.2	6.4	5.1	6.1	5.1	6.3	5.0	6.0	5.1	6.2	5.1	6.4	4.3	5.5	4.5	5.5	5.1	6.2
**BB (%DEI)**		1.0	1.8	1.0	1.7	1.1	1.8	0.9	1.7	1.1	2.0	1.1	1.8	1.0	1.6	1.0	1.8	0.9	2.0	0.6	1.1	1.0	1.8
**B (%DEI)**		15.4	7.7	15.4	7.4	15.7	7.2	15.6	7.1	15.9	7.3	15.5	7.3	15.7	7.2	15.2	7.4	14.3	7.1	14.1	7.0	15.4	7.3
**MM (%DEI)**		4.8	5.2	4.3	4.9	4.1	4.6	3.9	4.5	4.2	4.8	4.2	4.9	4.5	5.3	5.1	6.0	6.3	6.5	6.6	6.8	4.5	5.2
**L (%DEI)**		28.5	9.0	29.0	8.9	29.0	8.9	29.0	8.8	27.5	8.7	28.0	9.3	28.1	9.0	27.8	9.3	27.9	8.1	27.9	7.7	28.4	8.9
**T (%DEI)**		4.3	4.6	4.2	4.6	4.4	4.5	4.3	4.3	4.8	5.3	4.2	4.6	4.3	4.7	4.1	4.3	3.6	3.5	3.9	4.7	4.2	4.5
**D (%DEI)**		36.1	10.5	35.7	9.9	35.1	9.8	36.1	9.4	36.0	10.1	36.2	10.0	35.9	9.4	36.3	10.1	36.7	9.1	37.1	9.6	36.0	9.8
**E (%DEI)**		7.5	6.6	7.8	6.2	8.2	6.7	7.4	5.9	7.8	6.3	8.0	6.4	7.9	6.1	8.1	6.4	7.7	6.2	7.5	5.9	7.8	6.3
**U (%DEI)**		2.5	3.8	2.6	3.9	2.6	4.0	2.9	4.2	2.7	4.0	2.7	4.0	2.7	4.1	2.4	3.7	2.6	3.8	2.2	3.6	2.6	4.0
**Number of EDO >210 kJ**		4.56	0.90	4.63	0.93	4.70	0.94	4.63	0.91	4.68	0.99	4.62	0.92	4.64	0.92	4.67	0.91	4.67	0.88	4.65	0.92	4.64	0.92
**Number of EDO =<15% DEI**		1.88	0.93	1.96	0.98	2.02	0.98	1.95	0.96	1.97	1.05	1.94	0.96	1.95	0.96	2.00	0.95	1.97	0.89	1.96	0.94	1.96	0.96
**Number of EDO >15% DEI**		2.68	0.44	2.67	0.42	2.68	0.42	2.68	0.42	2.72	0.44	2.68	0.43	2.69	0.40	2.67	0.44	2.70	0.43	2.70	0.44	2.68	0.43
**Fast time (min)**		149	188	166	173	188	177	185	149	174	185	131	116	165	109	186	107	203	120	210	151	171	153
**Triglycerides (mmol/L)**		1.72	0.92	1.76	1.04	1.85	0.99	1.92	1.05	2.03	1.13	2.32	1.32	2.30	1.22	2.34	1.41	2.12	1.35	2.25	1.67	2.03	1.19
**Glucose (mmol/L)**		4.34	1.72	4.24	1.71	4.00	1.29	4.13	1.59	4.31	1.54	4.52	1.58	4.32	1.22	4.15	1.13	4.04	0.98	4.02	1.32	4.22	1.46
**Social Class**	non-manual	683	61%	827	60%	721	57%	752	63%	439	64%	655	60%	626	56%	592	58%	295	50%	123	51%	5713	59%

	manual	439	39%	556	40%	541	43%	450	37%	247	36%	435	40%	501	45%	424	42%	301	51%	117	49%	4011	41%
**Marital status**	married	974	87%	1242	90%	1107	88%	1034	86%	589	86%	957	88%	1004	89%	905	89%	540	91%	220	92%	8572	88%
	not married	148	13%	141	10%	155	12%	168	14%	97	14%	133	12%	123	11%	111	11%	56	9%	20	8%	1152	12%
		**8/9 (n 1122)**		**10 (n 1383)**		**11 (n 1262)**		**12 (n 1202)**		**13 (n 686)**		**14 (n 1090)**		**15 (n 1127)**		**16 (n 1016)**		**17 (n 596)**		**18/19 (n 240)**		**Men (n 9724)**	
**Education level**	no qualification/less O-level	290	26%	429	31%	415	33%	356	30%	216	32%	336	31%	335	30%	293	29%	158	27%	51	21%	2879	30%
	O-level/age 16 y	97	9%	117	9%	114	9%	116	10%	56	8%	96	9%	115	10%	76	8%	50	8%	25	10%	862	9%
	A-level/age 18 y	533	48%	625	45%	563	45%	544	45%	311	45%	499	46%	505	45%	477	47%	296	50%	117	49%	4470	46%
	Degree or equivalent	202	18%	212	15%	170	14%	186	16%	103	15%	159	15%	172	15%	170	17%	92	15%	47	20%	1513	16%
**Smoking status**	current	148	13%	142	10%	166	13%	128	11%	84	12%	131	12%	131	12%	109	11%	79	13%	31	13%	1149	12%
	Former	571	51%	762	55%	720	57%	683	57%	361	53%	576	53%	641	57%	559	55%	285	48%	120	50%	5278	54%
	never	403	36%	479	35%	376	30%	391	33%	241	35%	383	35%	355	32%	348	34%	232	39%	89	37%	3297	34%
**Physical activity**	inactive	348	31%	420	30%	430	34%	391	33%	206	30%	347	32%	340	30%	274	27%	121	20%	45	19%	2922	30%
	moderately inactive	264	24%	340	25%	314	25%	306	26%	175	26%	287	26%	260	23%	262	26%	126	21%	65	27%	2399	25%
	moderately active	264	24%	310	22%	257	20%	266	22%	168	25%	236	22%	284	25%	239	24%	160	27%	71	30%	2255	23%
	active	246	22%	313	23%	261	21%	239	20%	137	20%	220	20%	243	22%	241	24%	189	32%	59	25%	2148	22%
**Season of 1HC**	spring	315	28%	380	28%	339	27%	330	28%	174	25%	304	28%	336	30%	278	27%	162	27%	50	21%	2668	27%
	summer	283	25%	333	24%	293	23%	299	25%	158	23%	256	24%	299	27%	269	27%	141	24%	63	26%	2394	25%
	autumn	277	25%	355	26%	315	25%	304	25%	187	27%	281	26%	251	22%	258	25%	154	26%	75	31%	2457	25%
	winter	247	22%	315	23%	315	25%	269	22%	167	24%	249	23%	241	21%	211	21%	139	23%	52	22%	2205	23%
**Reporting status**	Low ER	221	20%	277	20%	229	18%	259	22%	139	20%	248	23%	213	19%	208	21%	103	17%	41	17%	1938	12%
	Acceptable ER	897	80%	1101	80%	1021	81%	933	78%	542	79%	836	77%	909	81%	801	79%	491	82%	196	82%	7727	80%
	High ER	4		5		12		10		5		6		5		7		2		3		59	

DEI, daily energy intake; 7dDD, 7-day diet diary; DEI, daily energy intake; BB, before breakfast, B, breakfast, MM, midmorning; L, lunch; T, tea; D, dinner; E, evening; U, unknown; AER, acceptable energy reporter; LER, low energy reporter; HER, high energy reporter.

Statistics are means and standard deviation for continuous variables and number and percent for categorical variables.

Supplement Table S2: Description of EPIC-Norfolk participants (WOMEN only) by hour at which their blood sample was taken at the first health examination (1993-1998).

	8/9 (n 1382)		10 (n 1720)		11 (n 1604)		12 (n 1437)		13 (n 917)		14 (n 1611)		15 (n 1357)		16 (n 1024)		17 (n 414)		18/19 (n 128)		All (n 11 594)	
**Age (y)**	58.2	9.4	59.4	9.6	59.6	9.3	60.1	9.3	59.3	9.3	59.0	9.0	58.5	9.0	56.6	8.8	52.7	6.5	51.6	6.2	58.6	9.3
**BMI (kg/m2)**	25.8	4.0	26.0	4.2	26.1	4.2	26.1	4.3	26.4	4.2	26.2	4.2	26.2	4.3	26.1	4.3	25.7	4.3	25.5	4.0	26.1	4.2
**Waist (cm)**	81	10	82	11	82	11	82	11	83	11	82	11	82	11	81	10	80	10	80	10	82	11
**DEI (MJ/d)**	7.2	1.7	7.2	1.7	7.2	1.6	7.2	1.6	7.2	1.7	7.2	1.6	7.2	1.6	7.2	1.7	7.3	1.6	7.2	1.5	7.2	1.6
**Protein (%DEI)**	15.7	2.8	15.7	3.0	15.9	3.0	15.9	2.9	15.8	2.8	15.8	2.9	15.9	2.9	15.8	3.0	15.6	3.0	15.6	3.0	15.8	2.9
**Fat (%DEI)**	33.2	5.7	33.2	5.8	33.1	5.6	33.1	5.6	33.2	5.8	33.3	5.6	33.3	5.6	32.9	5.6	33.1	5.7	31.7	6.0	33.2	5.7
**Carbohydrate (%DEI)**	47.9	6.5	47.8	6.3	47.6	6.2	47.8	6.2	47.6	6.4	47.8	6.2	47.9	6.1	47.9	6.4	47.5	6.3	47.6	7.0	47.8	6.3
**Alcohol (%DEI)**	3.1	4.6	3.3	4.8	3.3	4.8	3.1	4.4	3.3	4.8	3.1	4.4	2.9	4.1	3.3	4.7	3.9	4.9	5.2	6.5	3.2	4.6
**BB (%DEI)**	1.0	1.6	1.0	1.7	1.0	1.5	1.0	1.6	1.1	1.7	1.0	1.9	1.0	1.6	0.9	1.7	0.8	1.4	0.6	1.0	1.0	1.6
**B (%DEI)**	14.6	6.5	14.8	6.4	14.5	6.5	14.9	6.2	15.3	6.4	14.9	6.4	14.9	6.7	15.1	6.3	13.9	6.5	14.0	5.9	14.8	6.4
**MM (%DEI)**	3.8	3.6	3.5	3.6	3.5	3.6	3.6	3.5	3.7	3.7	3.6	3.4	3.7	3.6	3.9	3.9	4.5	4.4	4.1	3.6	3.7	3.6
**L (%DEI)**	29.1	8.5	29.3	8.8	29.5	8.5	29.1	8.0	28.0	8.4	28.5	8.3	28.4	8.6	28.1	8.2	27.5	7.7	27.4	8.6	28.8	8.4
**T (%DEI)**	4.8	4.6	4.4	4.2	4.7	4.5	4.7	4.4	4.7	4.7	4.8	4.7	4.8	4.6	4.5	4.2	4.5	4.1	4.3	3.8	4.7	4.4
**D (%DEI)**	36.9	9.9	36.7	9.4	36.6	9.6	36.7	9.4	37.3	9.5	37.3	9.9	37.3	9.2	38.0	9.3	39.6	9.9	39.6	11.3	37.1	9.6
**E (%DEI)**	7.3	5.7	7.5	5.9	7.4	5.8	7.2	5.5	7.2	5.9	7.1	5.3	7.1	5.8	7.0	5.5	6.7	5.6	7.3	6.4	7.2	5.7
**U (%DEI)**	2.6	3.5	2.8	3.8	2.9	3.9	2.9	3.8	2.8	4.0	2.9	4.0	3.0	4.0	2.6	3.5	2.7	3.5	2.8	4.7	2.8	3.8
**Number of EDO >210 kJ**	4.49	0.85	4.50	0.87	4.51	0.86	4.53	0.85	4.55	0.88	4.53	0.85	4.49	0.85	4.50	0.86	4.44	0.87	4.39	0.84	4.51	0.86
**Number of EDO =<15% DEI**	1.87	0.90	1.88	0.92	1.91	0.92	1.89	0.91	1.92	0.93	1.89	0.91	1.87	0.92	1.85	0.90	1.85	0.89	1.80	0.87	1.88	0.91
**Number of EDO >15% DEI**	2.62	0.40	2.62	0.40	2.60	0.40	2.64	0.42	2.63	0.41	2.63	0.41	2.62	0.39	2.64	0.40	2.59	0.42	2.59	0.46	2.62	0.41
**Fast time (min)**	146	183	168	169	188	175	187	173	150	171	132	128	160	114	178	112	200	149	179	123	166	157
**Triglycerides (mmol/L)**	1.40	0.78	1.50	1.03	1.50	0.79	1.59	0.90	1.66	0.96	1.75	0.98	1.82	1.08	1.73	1.03	1.59	0.92	1.43	0.76	1.61	0.95
**Glucose (mmol/L)**	3.97	1.49	3.88	1.07	3.90	1.08	3.98	1.22	4.51	1.46	4.59	1.54	4.44	1.40	4.18	1.11	4.02	1.06	4.03	1.06	4.16	1.32

**Social Class**	non-manual	864	63%	1030	60%	988	62%	907	63%	586	64%	985	61%	834	62%	630	62%	266	64%	89	70%	7179	62%
	manual	518	38%	690	40%	616	38%	530	37%	331	36%	626	39%	523	39%	394	39%	148	36%	39	31%	4415	38%
**Marital status**	married	1064	77%	1331	77%	1212	76%	1066	74%	711	78%	1306	81%	1044	77%	790	77%	331	80%	99	77%	8954	77%
	not married	318	23%	389	23%	392	24%	371	26%	206	23%	305	19%	313	23%	234	23%	83	20%	29	23%	2640	23%
		**8/9 (n 1382)**		**10 (n 1720)**		**11 (n 1604)**		**12 (n 1437)**		**13 (n 917)**		**14 (n 1611)**		**15 (n 1357)**		**16 (n 1024)**		**17 (n 414)**		**18/19 (n 128)**		**All (n 11 594)**	
**Education level**	no qualification/less O-level	541	39%	758	44%	704	44%	584	41%	371	41%	691	43%	587	43%	381	37%	107	26%	29	23%	4753	41%
	O-level/age 16 y	174	13%	193	11%	179	11%	166	12%	107	12%	206	13%	155	11%	123	12%	55	13%	12	9%	1370	12%
	A-level/age 18 y	529	38%	578	34%	554	35%	548	38%	353	39%	561	35%	481	35%	385	38%	143	35%	58	45%	4190	36%
	Degree or equivalent	138	10%	191	11%	167	10%	139	10%	86	9%	153	10%	134	10%	135	13%	109	26%	29	23%	1281	11%
**Smoking status**	current	143	10%	191	11%	180	11%	164	11%	101	11%	186	12%	152	11%	108	11%	62	15%	16	13%	1303	11%
	former	424	31%	585	34%	524	33%	453	32%	311	34%	535	33%	452	33%	292	29%	101	24%	44	34%	3721	32%
	never	815	59%	944	55%	900	56%	820	57%	505	55%	890	55%	753	56%	624	61%	251	61%	68	53%	6570	57%
**Physical activity**	inactive	417	30%	532	31%	501	31%	440	31%	248	27%	468	29%	360	27%	249	24%	101	24%	38	30%	3354	29%
	moderately inactive	449	33%	559	33%	490	31%	465	32%	297	32%	521	32%	459	34%	350	34%	141	34%	45	35%	3776	33%
	moderately active	293	21%	370	22%	383	24%	307	21%	211	23%	382	24%	321	24%	254	25%	107	26%	25	20%	2653	23%
	active	223	16%	259	15%	230	14%	225	16%	161	18%	240	15%	217	16%	171	17%	65	16%	20	16%	1811	16%
**Season of 1HC**	spring	382	28%	453	26%	439	27%	426	30%	206	23%	445	28%	383	28%	307	30%	116	28%	30	23%	3187	28%
	summer	384	28%	410	24%	400	25%	345	24%	252	28%	401	25%	336	25%	278	27%	102	25%	29	23%	2937	25%
	autumn	329	24%	438	26%	403	25%	342	24%	234	26%	403	25%	344	25%	245	24%	122	30%	43	34%	2903	25%
	winter	287	21%	419	24%	362	23%	324	23%	225	25%	362	23%	294	22%	194	19%	74	18%	26	20%	2567	22%
**Reporting status**	LER	324	23%	419	24%	367	23%	353	25%	240	26%	389	24%	351	26%	277	27%	100	24%	30	23%	2850	25%
	AER	1056	76%	1297	75%	1232	77%	1079	75%	671	73%	1215	75%	1003	74%	742	73%	313	76%	98	77%	8706	75%
	HER	2		4		5		5		6		7		3		5		1		0		38	

DEI, daily energy intake; 7dDD, 7-day diet diary; DEI, daily energy intake; BB, before breakfast, B, breakfast, MM, midmorning; L, lunch; T, tea; D, dinner; E, evening; U, unknown; AER, acceptable energy reporter; LER, low energy reporter; HER, high energy reporter.

Statistics are means and standard deviation for continuous variables and number and percent for categorical variables.

Supplement Table S3: Characteristics of EPIC-Norfolk participants by energy reporting status.

The high energy reporters (n=59 men, n=38 women) were excluded from this analysis. In both men and women, low energy reporters (LER) compared to acceptable energy reporters (AER) had a higher BMI and waist circumference. DEI was approximately 2.5-3 MJ/d lower among LER; however, their %DEI reported at lunch and dinner was higher as was the %DEI derived from protein. LER reported a lower frequency of EDO, which was more driven by a lower EDO<15%DEI, than a lower frequency of EDO>15%DEI.

The length of time fasted at the time of the health examination was higher among LER, although triglyceride concentrations (but not glucose) were higher.

LER reported more often being active and less often being inactive. Reporting status was associated with socio-demographics, such that manual social class and education below O-level were more common among LER.

	Men (n 9665)				Women (n 11,556)			
	LER (n 1938)		AER (n 7727)		LER (n 2850)		AER (n 8706)	
**Age (y)**	59.61	9.27	59.41	9.30	58.11	8.86	58.79	9.39
**BMI (kg/m2)**	28.31	3.59	26.01	2.99	27.96	4.77	25.45	3.82
**Waist circumference (cm)**	100.5	10.11	94.34	9.03	85.55	11.65	80.43	9.87
**DEI (MJ/d)**	6.91	1.24	10.12	1.78	5.36	0.93	7.78	1.29
**Protein (% Energy)**	16.71	2.97	14.54	2.20	17.67	3.49	15.19	2.41
**Fat (% Energy)**	32.02	5.93	33.87	5.19	30.99	6.28	33.85	5.27
**Carbohydrate (% Energy)**	46.23	7.35	46.46	6.46	48.48	7.03	47.56	6.00
**Alcohol (% Energy)**	4.99	6.44	5.10	6.10	2.78	4.68	3.35	4.61
**BB (%DEI)**	1.05	2.05	0.97	1.66	1.01	1.86	0.98	1.56
**B (%DEI**	15.07	8.08	15.53	7.08	14.59	6.97	14.83	6.22
**MM (%DEI)**	3.73	5.16	4.72	5.19	3.23	3.79	3.80	3.57
**L (%DEI)**	28.90	11.03	28.29	8.29	29.44	9.60	28.56	8.00
**T (%DEI)**	3.79	4.95	4.33	4.39	4.02	4.68	4.87	4.35
**D (%DEI)**	38.36	12.14	35.42	9.03	38.78	11.2	36.62	8.92
**E (%DEI)**	6.71	6.17	8.08	6.33	6.52	6.04	7.42	5.50
**U (%DEI)**	2.40	3.83	2.66	3.99	2.43	3.66	2.91	3.82
**Number of recording sections**	5.78	1.01	6.19	0.88	6.10	0.93	6.44	0.84
**Number of EDO >210 kJ**	4.06	0.86	4.78	0.88	3.98	0.75	4.67	0.82

		Men (n 9665)				Women (n 11,556)			
		LER (n 1938)		AER (n 7727)		LER (n 2850)		AER (n 8706)	
**Number of EDO<15%DEI**		1.49	0.86	2.07	0.95	1.39	0.78	2.04	0.89
**Number of EDO>15%DEI**		2.58	0.46	2.71	0.41	2.58	0.42	2.64	0.40
**Fast time (min)**		187	178	167	146	183	184	160	147
**Triglycerides (mmol/L)**		2.18	1.34	1.99	1.15	1.67	0.97	1.59	0.94
**Glucose (mmol/L)**		4.27	1.53	4.21	1.45	4.16	1.31	4.15	1.32
**Social Class**	non-manual	1081	55.8%	4611	59.7%	1577	55.3%	5582	64.1%
	manual	857	44.2%	3116	40.3%	1273	44.7%	3124	35.9%
**Marital status**	married	1688	87.1%	6840	88.5%	2219	77.9%	6710	77.1%
	not married	250	12.9%	887	11.5%	631	22.1%	1996	22.9%
**Education level**	no qualification/ less O-level	644	33.2%	2212	28.6%	1367	48.0%	3368	38.7%
	O-level/age 16 y	165	8.5%	688	8.9%	292	10.2%	1073	12.3%
	A-level/age 18 y	852	44.0%	3596	46.5%	961	33.7%	3217	37.0%
	Degree or equivalent	277	14.3%	1231	15.9%	230	8.1%	1048	12.0%
**Smoking status**	current	245	12.6%	888	11.5%	368	12.9%	923	10.6%
	former	1083	55.9%	4167	53.9%	956	33.5%	2759	31.7%
	never	610	31.5%	2672	34.6%	1526	53.5%	5024	57.7%
**Physical activity**	inactive	507	26.2%	2389	30.9%	680	23.9%	2652	30.5%
	moderately inactive	514	26.5%	1878	24.3%	961	33.7%	2811	32.3%
	moderately active	424	21.9%	1816	23.5%	579	20.3%	2066	23.7%
	active	493	25.4%	1644	21.3%	630	22.1%	1177	13.5%
**Season**	spring	553	28.5%	2096	27.1%	830	29.1%	2342	26.9%
	summer	512	26.4%	1866	24.1%	771	27.1%	2157	24.8%
	autumn	428	22.1%	2015	26.1%	654	22.9%	2242	25.8%
	winter	445	23.0%	1750	22.6%	595	20.9%	1965	22.6%
**Hour of blood sample**	8/9	221	11.4%	897	11.6%	324	11.4%	1056	12.1%
	10	229	14.3%	1101	14.2%	419	14.7%	1297	14.9%
	11	277	11.8%	1021	13.2%	367	12.9%	1232	14.2%
	12	259	13.4%	933	12.1%	353	12.4%	1079	12.4%
	13	139	7.2%	542	7.0%	240	8.4%	671	7.7%
	14	248	12.8%	836	10.8%	389	13.6%	1215	14.0%
	15	213	11.0%	909	11.8%	351	12.3%	1003	11.5%
	16	208	10.7%	801	10.4%	277	9.7%	742	8.5%
	17	103	5.3%	491	6.4%	100	3.5%	313	3.6%
	18/19	41	2.1%	196	2.5%	30	1.1%	98	1.1%

7dDD, 7-day diet diary; LER, low energy reporter; AER, acceptable energy reporter; DEI, daily energy intake; EDO, eating and drinking occasion (any type); EDO>15%DEI, EDO containing more than 15%DEI; EDO<15%DEI, EDO containing less than 15%DEI.

Statistics are means and standard deviation for continuous variables and number and percent for categorical variables.
